# Locked-In Syndrome after Traumatic Basilar Artery Entrapment within a Clivus Fracture: A Case Report and Review of the Literature

**DOI:** 10.1089/neur.2020.0015

**Published:** 2020-09-14

**Authors:** Tjerk J. Lagrand, Vincent A.J. Bruijnes, A.M. Madeleine Van der Stouwe, Eric A. Deckers, Aryan Mazuri, Bram Jacobs

**Affiliations:** ^1^ Department of Neurology, University of Groningen, University Medical Center Groningen, Groningen, the Netherlands.; ^2^ Department of Radiology, University of Groningen, University Medical Center Groningen, Groningen, the Netherlands.; ^3^ Department of Surgery, University of Groningen, University Medical Center Groningen, Groningen, the Netherlands.

**Keywords:** basilar artery, clivus fracture, entrapment, head trauma, locked-in syndrome

## Abstract

We report the case of a 58-year-old male with a rare vascular complication after traumatic head injury: entrapment of the basilar artery into a fracture of the clivus, ultimately leading to a locked-in syndrome due to brainstem infarction. Review of the literature revealed 19 earlier published cases of basilar artery entrapment within traumatic longitudinal clival fractures. In the majority of these patients there is an unfavorable neurological outcome.

## Introduction

Clival fractures are rarely seen in patients after head trauma, but when present they are associated with cranial nerve palsies, cerebrospinal fluid (CSF) leaks, and vascular injuries of the anterior and posterior circulation.^1^ Further, clivus fractures are associated with subarachnoid hemorrhage, arterial dissections, and stroke, resulting in high morbidity and mortality rates.^2^ Here, we report an unusual case of entrapment of the basilar artery within a longitudinal fractured clivus, which caused pontine and midbrain infarction leading to a locked-in syndrome in a 58-year-old patient.

## Case Report

**FIG. 1.**
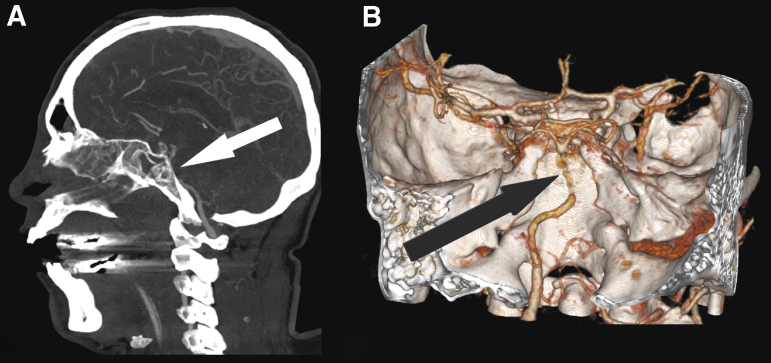
Computed tomography images. **(A)** Sagittal maximized intensity projection (MIP) image showing basilar artery herniation into the clivus fracture and its trans-sinussoidal course. **(B)** Three-dimensional (3D) reconstruction of the skull base and the circle of Willis' region displaying the basilar artery entrapment

A previously healthy 58-year-old male was found unconscious next to his bike in a pit excavated for sewer maintenance along the road (depth of approximately 1.5 m). On his initial assessment the ambulance service physician found a Glasgow Coma Scale score of 12 (E3M6V3), left frontal cephalohematoma, periorbital ecchymosis, and rhinorrhea. His pupils were 3 mm bilaterally with dubious light reflexes and intact corneal responses. The patient was sedated and intubated for safe transport and sequentially admitted to our Emergency Department (ED). Contrast-enhanced computed tomography (CT) scanning of his head and neck showed extensive traumatic subarachnoid hemorrhaging, a left-sided subdural hematoma, and an unusual course of the basilar artery with anterior deviation into the posterior aspect of the left sphenoid sinus that was filled with blood due to the extensive sinonasal fractures. (Fig. 1A). Three-dimensional (3D) reconstructions revealed that the basilar artery herniation occurred through a longitudinal clival fracture (Fig. 1B). Cranially, the basilar artery exited the left sphenoid sinus through the posterior wall and continued within the basal cisterns. In addition, there were extensive skull base fractures including the right carotid canal, hard palate, and pterygoid palates of the sphenoid bone. Moreover, there were fractures of the inferior walls of both orbits, left zygoma, and a small ventral pneumothorax.

**FIG. 2.**
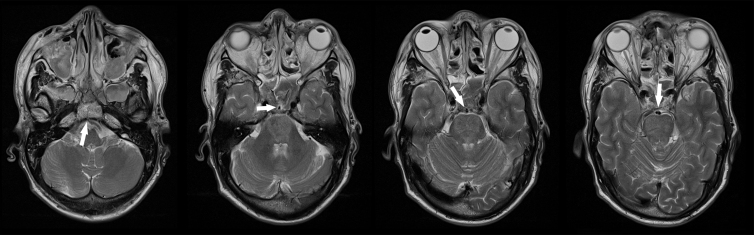
Axial magnetic resonance images. Sequential T2-weighted images (from caudal toward cranial) of the basilar artery pathway showing herniation of the artery into the clivus fracture and the trans-sinussoidal course through the left sphenoidal sinus.

**FIG. 3.**
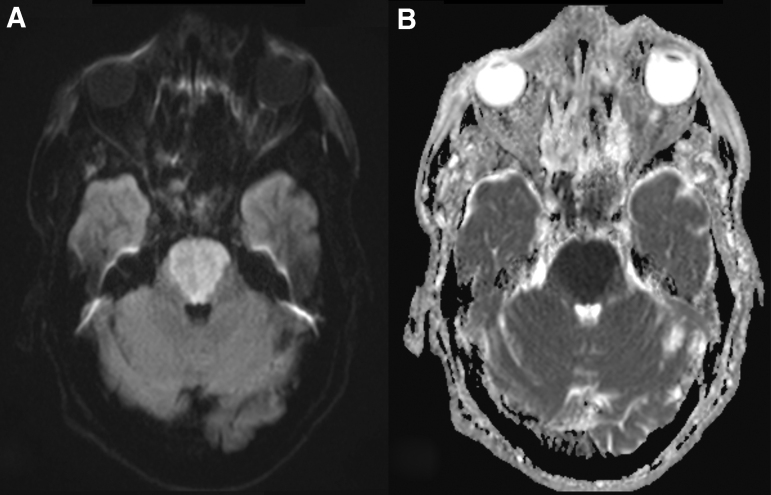
Axial magnetic resonance images. **(A)** Diffusion-weighted image (DWI) confirms acute pontine ischemia with **(B)** corresponding apparent diffusion coefficient (ADC) image.

After initial surgical stabilization according to the Advanced Trauma Life Support (ATLS) guidelines, the patient was transferred to the intensive care unit (ICU) for further supportive care and monitoring. Later on the day of admission, after withdrawal of sedatives, he was breathing spontaneously but appeared quadriplegic and anartric. The patient was able to respond adequately with vertical eye movements only and therefore was diagnosed with a locked-in syndrome. On hospital day 2, magnetic resonance imaging (MRI) of the brain confirmed the herniation of the basilar artery (Fig. 2) and showed severe mesencephalic and pontine ischemia (Fig. 3). No antiplatelet or anticoagulant therapy was administered because of his intracranial and sinonasal hemorrhages. The patient remained respiratory and hemodynamically stable for 3 weeks in the ICU, without significant neurological improvement. A tracheostomy was performed and after 3 more weeks he became able to cough strongly and effectively, and reflexive swallowing was present. At the time of writing, 2 months after his head injury, the patient is in the medium care unit of the department of neurology, completely dependent for activities of daily living (ADL) and pending transfer to a neurorehabilitation center.

## Discussion

Brainstem infarction as a result of basilar artery entrapment within a clivus fracture is a rare vascular complication after head trauma. The clivus, a backward sloping bony process, is located at the midline in the deepest part of the skull base. It is partly composed of the sphenoid and occipital bone, and on the axial plane it is located just caudally and posterior of the sphenoid sinuses. Due to this anatomical localization, traumatic fractures of the clivus are rare, occurring mainly in patients with severe and complex head injury. In the literature, the incidence of clivus fractures varies between 0.21% and 1.2% in cases of head trauma.^1,3^ Based on their orientation, clivus fractures can be classified radiographically into three types: longitudinal, transverse, and oblique.^4^ The extent and type of fracture depends on the trajectory and magnitude of the trauma. Oblique and transverse fractures are associated with damage to the anterior cerebral circulation and multiple cranial nerve deficits.^5^ This might be due to increased shearing forces on these nerves by the trauma or by direct compression of fractured bony structures. Longitudinal fractures are associated with high mortality (up to 41%) and poor neurological outcome, due to their association with damage to the vertebrobasilar arteries. In particular, local compression of the basilar artery, dissections, and subarchnoidal hemorrhage are described.^3^

The proposed trauma mechanism of basilar artery incarceration consists of three components. First is vertical compression that leads to a longitudinal linear clivus fracture with dura laceration. Second is inertia of the basilar artery by brainstem and cerebellar mass in forward direction, and third is fracture closing, which inserts the basilar artery and causes stenosis, dissection, or occlusion. This results in brainstem and/or cerebellar infarction, depending on anatomical variations, location of the fracture, and the side of arterial entrapment.^6^

The diagnosis of basilar artery herniation can be made by a combination of radiological modalities. In the acute situation, contrast-enhanced CT can quickly be performed to show fractures of the skull base and CT-angiography can demonstrate the exact site of basilar artery entrapment. Correct interpretation is important, because a misdiagnosis of basilar thrombosis and attempted endovascular intervention is easily made.^7^ 3D reconstructions can illustrate the exact course of the basilar artery extending through the fracture. Follow-up MRI of the brain can demonstrate infarction occurring within the vertebrobasilar vascular territory, which includes the brainstem, cerebellum, midbrain, thalami, and several areas of temporal and occipital lobes.

To our knowledge, in 1964 the first case of traumatic entrapment of the basilar artery into a fracture line of the clivus leading to herniation into the sphenoid sinus was reported.^8^ As a result, the patient described developed severe brainstem and cerebellar infarction and died 14 days after his injury due to respiratory and cardiac failure. A PubMed search revealed 18 additional cases of traumatic basilar artery entrapment within a longitudinal clivus fracture worldwide.^6,7,9–24^ Interestingly, all of the patients were men. Outcomes in these patients were poor: 8 died, 1 remained in a vegetative state, 3 developed a locked-in syndrome, 5 were left with mild to severe paresis, and only 1 patient recovered completely. Although standard treatment has not been established yet, there are some patients in whom antithrombotic treatment was initiated with eventually some improvement of ADL. On the other hand, one should be cautious with antithrombotic therapy in trauma patients. In patients who deteriorate because of thrombosis due to basilar artery dissection and/or stenosis, there might be a place for endovascular treatment. However, no cases have been described so far. In our patient, neurosurgical or endovascular treatment was discussed in the ED, but the risk of these invasive treatments was considered to be too high. After follow-up imaging we decided not to start antithrombotic therapy, because of his intracranial hemorrhages and other systemic injuries.

This case report describes an uncommon traumatic complication of a clivus fracture, with basilar artery herniation into the sphenoid sinus. Subsequently, our patient developed a locked-in syndrome, a catastrophic neurological condition in which patients are conscious and aware, but cannot move or speak due to complete paralysis of nearly all voluntary muscles in the body except for vertical eye movements and blinking. Patients with a locked-in syndrome due to traumatic basilar artery entrapment are rarely reported in the literature.

## Abbreviations Used

3D: three-dimensional
ADC: apparent diffusion coefficient
ADL: activities of daily living
ATLS: Advanced Trauma Life Support
CSF: cerebrospinal fluid
CT: computed tomography
DWI: diffusion-weighted image
ED: Emergency Department
ICU: intensive care unit
MIP: maximized intensity projection
MRI: magnetic resonance imaging
